# The effect of HIV infection on the incidence and severity of circular external fixator pin track sepsis: a retrospective comparative study of 229 patients

**DOI:** 10.1007/s11751-014-0194-y

**Published:** 2014-07-24

**Authors:** Nando Ferreira, Leonard Charles Marais

**Affiliations:** 1 Tumour Sepsis and Reconstruction Unit, Department of Orthopaedic Surgery, Greys Hospital, Nelson R. Mandela School of Medicine, University of KwaZulu-Natal, Pietermaritzburg, 3201 South Africa; 2 Present Address: Department of Orthopaedic Surgery, Greys Hospital, Pietermaritzburg, KwaZulu-Natal 3201 South Africa

**Keywords:** HIV, Pin track sepsis, Complication, Ilizarov, Circular external fixator

## Abstract

Pin track sepsis is a common complication of circular external fixation. HIV status has been implicated as an independent risk factor for the development of pin track infection and has been cited as a reason not to attempt complex limb reconstruction in HIV-positive patients. This retrospective review of patients treated with circular external fixators looked at the incidence of pin track sepsis in HIV-positive, HIV-negative and patients whose HIV status was unknown. The records of 229 patients, 40 of whom were HIV-positive, were reviewed. The overall incidence of pin track sepsis was 22.7 %. HIV infection did not affect the incidence of pin track sepsis (*p* = 0.9). The severity of pin track sepsis was not influenced by HIV status (*p* = 0.9) or CD_4_ count (*p* = 0.2). With the employment of meticulous pin insertion techniques and an effective postoperative pin track care protocol, circular external fixation can be used safely in HIV-positive individuals.

## Introduction

 External fixation, and circular external fixation in particular, has evolved as an indispensible component of contemporary trauma and limb reconstruction surgery. Owing to its minimally invasive nature, circular fixators are being used increasingly in the management of skeletal trauma. In injuries associated with soft tissue compromise, such as periarticular fractures of the tibia, circular fixation has been shown to decrease the incidence of deep infection [1–6]. Its use is well established in the reconstruction of post-traumatic, post-infective bone defects and congenital deformities. This treatment modality is, however, associated with its own set of complications of which the most frequent is pin track sepsis with the reported incidences ranging from 11.3 to 100 % [4, 7–15].

Pin track sepsis is often the first clinical manifestation of a vicious cycle of pin loosening and sustained pin site infection. It is a misconception that pin track sepsis result in pin loosening; pin loosening is more often the inciting event that leads to pin site infection [14, 16–19]. Failure of the pin–bone interface can have catastrophic consequences and may lead to failure of the reconstruction and, ultimately, limb ablation in some. A meticulous approach to pin and wire insertion combined with a structured protocol of pin site care has been shown to decrease the incidence of pin track sepsis [4, 20, 21]. Certain patient factors may, however, influence the incidence and severity of pin track sepsis. Poor diabetic control and HIV infection have both been implicated as independent risk factors for the development of pin track infection [7, 15, 22–24].

HIV infection was previously considered to be a relative contraindication for the use of external fixators. A recent study from Malawi investigating the use of monolateral external fixators in tibial trauma found an increased incidence and severity of pin track sepsis in HIV-positive patients [22–24]. This study is cited frequently against limb reconstruction with external fixation in HIV-positive patients. The use of circular fixators, in particular, has been avoided in HIV-positive patients due to the prolonged periods of treatment required.

South Africa has the highest incidence of HIV infection in the world. The 2011 National Antenatal Sentinel Survey reported a national prevalence of 17.3 %, with areas like KwaZulu-Natal approaching 25 % [25]. The majority of these patients are between 20 and 50 years old. South Africa also has one of the highest incidences of road traffic accidents in the world, affecting mostly young adults [26, 27]. The HIV pandemic in South Africa, combined with the high incidence of trauma, has resulted in many HIV-positive patients requiring treatment for complex trauma or a need for post-traumatic limb reconstruction. Of note is that the overall fracture prevalence is increased in HIV-positive compared to HIV-negative patients [28–30].

This retrospective review aims to compare the rate and severity of pin track sepsis in HIV-positive and HIV-negative patients treated with circular external fixators. The research proposal was reviewed and approved by the local ethics committee. An extensive literature review revealed this current study to be the largest yet to compare the incidence of pin track sepsis in HIV-positive and HIV-negative patients. It is currently also the only study investigating the effect of HIV infection on the incidence and severity of pin track sepsis with the use of circular external fixators.

## Materials and methods

The study population consisted of all patients who were treated with circular external fixators at our institution between July 2008 and December 2012. Patients were included if they had completed treatment and had the external fixator removed. Patients were excluded if the external fixator was not applied at our institution or if the records were insufficient for the required data.

All patients were offered voluntary HIV counseling and testing. The CD_4_ count of all HIV-positive patients was measured. Patients with CD_4_ counts below 350 cells/mm^3^ were started on highly active antiretroviral therapy (HAART) in accordance with South African national antiretroviral treatment guidelines.

The fixator design and application followed the general principles as outlined by Catagni with the emphasis on construction of a stable frame configuration [31–36]. Particular attention was paid to atraumatic pin and wire insertion. Recognized anatomical safe zones were used and insertion was carried out with as little heat and energy transfer as possible [31, 36, 37]. Postoperative pin track care followed the protocol previously set out by Ferreira and Marais [21]. Outpatient follow-up was scheduled at two to four weekly intervals until frame removal. At every clinic visit, the progress was assessed and any complications, including pin track sepsis, were documented. Pin site infections were graded according the Checketts and Otterburn classification (Table 1) [38].

**Table 1:** Checketts–Otterburn classification

Grade	Characteristics	Treatment
Minor infection		
1	Slight redness, little discharge	Improved pin site care
2	Redness of the skin, discharge, pain and tenderness in the soft tissue	Improved pin site care, oral antibiotics
3	Grade 2 but no improvement with oral antibiotics	Affected pin or pins resited and external fixation can be continued
Major infection		
4	Severe soft tissue infection involving several pins, sometimes with associated loosening of the pin	External fixation must be abandoned
5	Grade 4 but radiographic changes	External fixation must be abandoned
6	Infection after fixator removal. Pin track heals initially, but will subsequently break down and discharge in intervals. Radiographs show new bone formation and sometimes sequestra	Curettage of the pin tract

A retrospective review was undertaken and the variables recorded included patient demographics, HIV status, CD_4_ count and use of antiretroviral medication, indications for circular fixation, type of external fixator used, pin track complications and treatment of these complications. Results were analyzed using the independent *t* test, one-way ANOVA test and the Kruskal–Wallis H test to ascertain whether HIV infection had any effect on the incidence or severity on pin track sepsis.

## Results

The records of 274 patients were reviewed. Forty-five patients were excluded because the external fixators had not yet been removed. Therefore, 229 patients (163 males and 66 females) were included: The mean age was 34.5 years (standard deviation ± 15.4, range 6–71 years); mean time in external fixation was 22.9 weeks (SD ± 14.7, range 6–104 weeks).

The external fixators applied consisted of 71 Ilizarov fixators (Smith and Nephew, Memphis, TN), 91 Truelok fixators (Orthofix, Verona, Italy), 65 Taylor Spatial Frames (Smith and Nephew, Memphis, TN) and two TL-Hex fixators (Orthofix, Verona, Italy) (Table 2). The indications for the use of the external fixators are listed in Table 3.

**Table 2:** External fixators applied

	HIV+	HIV−	Unknown	Total
Ilizarov	14	44	13	71
Truelok	21	65	5	91
Taylor Spatial Frame	5	57	3	65
TL-Hex	0	2	0	2
Total	40	168	21	229

**Table 3:** Circular external fixator indications

Indications	HIV+	HIV−	Unknown
Complex trauma	7	21	3
Periarticular fracture	17	50	12
Non-union	5	25	2
Bone transport	1	7	1
Bone defect	2	3	
Limb lengthening		1	
Chronic osteomyelitis	3	5	
Deformity correction	5	56	3
Total	40	168	21

The patients were divided into groups according to their HIV status. A third group was made up of patients who refused HIV testing and designated as the unknown group. The HIV-positive group consisted of 40 (17.5 %) patients. The mean age was 37.2 years (SD ± 10.2, range 8–56 years). Time in the external fixator averaged 26 weeks (SD ± 16.6, range 6–77 weeks). The HIV-negative group consisted of 168 (73.4 %) patients. The mean age was 33.2 (SD ± 16.5, range 6–71 years) and time in the external fixator averaged 33.2 weeks (SD ± 16.5, range 6–71 weeks). The group whose HIV status was unknown consisted of 21 (9.2 %) patients. Their mean age was 39.7 years (SD ± 13.1, range 17–59 years) and time in external fixation averaged 18.9 weeks (SD ± 10.2, range 7–50 weeks). There was no statistically significant difference between the three groups in terms of age (*p* = 0.09) or time in the external fixator (*p* = 0.18).

**Fig. 1:**
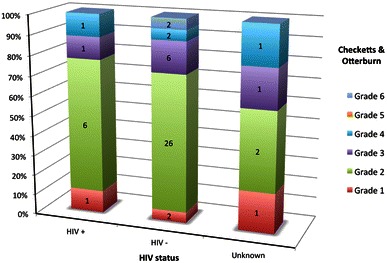
Pin track infection grades in HIV+, HIV− and Unknown groups

Pin track infection occurred in 52 (22.7 %) out of 229 patients. In the subgroups, nine (22.5 %) patients in the HIV-positive group (*n* = 40), 38 (22.6 %) patients in the HIV-negative group (*n* = 168) and five (23.8 %) patients in the unknown group (*n* = 21) developed pin track sepsis. Checketts and Otterburn grades for the three groups are shown in Fig. 1. There was no statistically significant difference in the incidence of pin track sepsis between the three groups (*p* = 0.94). Furthermore, the three groups had no statistically significant differences in terms of severity of pin track sepsis (*p* = 0.9).

A subgroup analysis of the HIV-positive patients (*n* = 40) was undertaken. Mean CD_4_ count was 347.4 cells/mm^3^ (D ± 162.4, range 82–682 cells/mm^3^) and 25 (62.5 %) patients were receiving HAART. Our data showed that CD_4_ count had no influence on either the incidence (*p* = 0.57) or severity (*p* = 0.21) of pin track sepsis in the HIV-positive group.

## Discussion

Pin track sepsis remains a common complication with the use of external fixators [7, 15]. Quoted incidences range from 11.3 to 100 % [9–13]. Mostafavi reported a 71 % incidence of pin site infection in reconstructive surgery [11].

The use of meticulous pin insertion techniques and the implementation of an evidence-based pin track care protocol can reduce the incidence of pin track sepsis with circular external fixation in reconstructive surgery to approximately 25 % [4]. Our results compare favorably to previously published figures with an overall pin track sepsis incidence of 22.7 % (52 out of 229) observed in this series.

Several factors have been implicated in the development of pin track sepsis [4, 21]. They include frame design and biomechanics, pin and wire insertion techniques, point of commencement of pin track care and the specific care protocol employed [7, 8, 12, 13, 40]. Strategies to reduce pin track sepsis should include measures aimed at optimization of these factors. Some non-modifiable risk factors have also been associated with pin site infection. These include diabetes mellitus and HIV infection [7, 15, 22–24].

HIV infection has prompted many orthopedic and trauma surgeons to avoid the use of circular external fixators for the purpose of limb reconstruction in HIV-positive patients. Norrish and Harrison published the first data comparing pin track infection with the use of monolateral external fixators in HIV-positive and HIV-negative patients [22, 24, 39]. They reported on 13 HIV-positive and 34 HIV-negative patients and found significantly more infections requiring pharmaceutical or surgical intervention in the HIV-positive group. Our results differ in that we could show no correlation between the incidence or severity of pin track sepsis and HIV status. Our results do correlate with the findings of no correlation between CD_4_ count and the severity of pin track infection in HIV-positive patients. The low patient numbers and wide CD_4_ range could explain the apparent lack of relationship and more research is required.

In conclusion, while pin track sepsis is a common complication with the use of circular external fixators, we did not find that the incidence or severity of pin track sepsis was influenced by HIV infection or degree of immune compromise. This finding should not preclude the use of circular external fixators for complex trauma and limb reconstruction in HIV-positive individuals.
